# Policy proposals for a new welfare: the development of the family and community nurse in Italy as the key to promote social capital and social innovation

**DOI:** 10.1017/S146342361800083X

**Published:** 2019-06-28

**Authors:** Silvia Marcadelli, Alessandro Stievano, Gennaro Rocco

**Affiliations:** 1 Dept of Biomedicine and Prevention - Doctoral Degree in Nursing Sciences Tor Vergata University, Rome, Italy; 2 Research coordinator Centre of Excellence for Nursing Scholarship, OPI, Rome, Italy; 3 Director Centre of Excellence for Nursing Scholarsip, OPI, Rome, Italy; 4 Schools of Health Professional, Catholic University Our Lady of Good Counsel, Tirana, Albania

**Keywords:** community health nursing, family health nursing, primary health care, social capital, social innovation

## Abstract

**Aim:**

To discuss the development of the family and community health nurse (FCHN) in Italy by focusing on three levels: organisational, political and theoretical.

**Background:**

The role of the FCHN in Italy is not yet embedded evenly across the Italian National Health System (INHS) and does not have formal recognition, either contractually or organisationally. Although complementary post-basic training has been available for over a decade, the FCHN’s role in Italy currently exists only in pilot form. In some regions, the FCHN has operated for longer, thanks to which a clearer understanding of the functions and responsibilities required by the FCHN has emerged. Proposals for professional and social policies have emerged, as the FCHN’s role may be an answer to health problems and a contributor to the construction of social capital, capable of influencing both individual and collective well-being.

**Methods:**

A mixed method investigation via a parallel concurrent design to identify the organisational models for the FCHN was conducted across Italy. In this paper, two profiles are discussed – family and community health nursing and FCHN – but each with its different connotations. The former refers to the practice of nursing and the latter to the nursing practitioners working with family and the community.

**Conclusion:**

We describe the expected future outcomes for FCHNs as elements of social innovation for the development of a new welfare system.

## Introduction

The new European health policy framework – Health 2020, issued by the World Health Organization (WHO) – identifies primary health care (PHC) as a cornerstone of health systems and as a key factor for addressing welfare challenges.

According to the WHO European region (Büscher *et al.*, 2009), nurses are the most important group providing health care in European communities. Despite recognition of the family health nurse (FHN) as a key figure in PHC and a professional capable of making a very substantial contribution to health promotion and disease prevention, in addition to being a caregiver (WHO, 1999), PHC in Italy has traditionally been managed by physicians. This model is similar to other European countries like Germany (Kendall and Bryar, 2017). Moreover, the Italian welfare system is identified as a ‘familistic’ one, at least substantially if not formally (Ascoli and Ranci, 2002; Saraceno, 2002; Ranci, 2004; Ferrera, 2006), because social protection policies have developed slowly and residually with the implicit idea that families bear responsibilities in providing care to patients (Vicarelli and Bronzini, 2009).

In Italy, while the number of medical personnel is comparatively large, there is still a chronic lack of nursing staff. As reported by the Organisation for Economic Co-operation and Development (OECD), with 3.8 practising physicians per 1000 people, Italy has one of the highest ratios of physicians in the OECD countries; and yet, the proportion of nurses is considerably lower, at 6.4 per 1000 people. The nurse-to-doctor ratio (1.4) is one of the lowest in the OECD Countries, where the average is 2.8 nurses to every doctor (OECD, 2017). Consequently, there are not enough nurses in PHC services. A major change in nurse training took place in Italy between 1999 and 2011 (Rocco *et al*., 2014), and today advanced educational programmes are available; unfortunately, these are not always useful for promoting career development. The first post-basic training programme in family and community nursing started in 2004. Despite this, the family and community health nurse (FCHN) still lacks specific contractual or organisational recognition from the Italian health system (Marcadelli and Bertolazzi, 2017).

Nonetheless, Italian local authorities have significant autonomy in health policies, and so several regions have introduced, in pilot form or via local health laws (or at least regulated at the organisational level), a role for FCHNs.

Interestingly, while at the international level there are different terms used to describe the different role or advanced role in nursing, in Italy this broad category is subdivided into two branches: ‘Paediatric Nurses’ and ‘Nurses’. The terms ‘Family Health Nurses’ and ‘Community Health Nurses’ are used to identify specific areas of nurse intervention without any statutory recognition, and often, in the Italian context, both expressions – family and/or community nurse – can be found. But political and/or organisational decisions are made based on the distinction between these two expressions identifying nurses’ work in primary care. For the sake of clarity, we reserve the use of the abbreviation FCHN for the nurse practitioners and refer to the practice (aka, system or model) in the full form: ‘family and community health nursing’.

In this paper, we present a brief overview of the current situation of the family and community health nursing in Italy, focusing on three levels: organisational, political and theoretical. We conclude by stating the expected future outcomes for FCHNs as elements of social innovation for the development of a new welfare system.

## About the Italian primary care system

The Italian National Health Service (INHS) has been public since 1978, guaranteeing equal care and rights for all citizens. The system is organised into three levels – national, regional and local – and provides universal cover. The national level sets the goals and guiding principles of the health system, allocates funding to regions and sets the standard and the minimum healthcare provisions available nationwide (Livelli Essenziali di Assistenza – LEA). At the regional level, the regions are responsible for organising and delivering health care. At the local level, geographically based local health authorities (Aziende Sanitarie Locali – ASL) monitor public health and deliver community health services, primary care, and secondary and specialist care either directly or through public hospitals or accredited private providers (Cicchetti and Gasbarrini, 2016). The national level defines general policies targeting health prevention and health improvement, while the regions, through their own health departments and ASLs, implement public health policies. These organisations are responsible for protecting the population’s health, promoting health, preventing diseases and disabilities, and improving quality of life (Ferré et al., 2014). They provide preventative medicine and public health services, primary care (including family medicine and community services, such as primary medical and nursing care, home care for the elderly and disabled, hospice care) and secondary care. Each ASL is divided into districts that directly control the supply of public health and primary care. General practitioners (GPs) and paediatricians provide family medicine services that play a gatekeeping role and represent the first point of contact with the healthcare system. They are responsible for prescribing medications and making initial diagnoses of health problems; they can then refer patients for specialist consultations or further levels of care if required (Fattore *et al.*, 2009; Caputo, 2013). Although they work for the regional health system, they are not employees of the ASL as their work is contracted through a national agreement (Petrazzuoli, 2016). The majority of GPs and paediatricians work alone (solo practice model) in their own surgeries, although national contracts and regional agreements encourage group practices by offering them extra income and extra resources, including nursing and staff support (Ferré *et al.*, 2014; Petrazzuoli, 2016). In recent years, efforts have been made to reorganise the primary care system with the objective of shifting from the traditional solo practice model to an integrated care model that links different healthcare professionals (Fattore *et al.*, 2009).

## Nurses in Italy

In 2001, training programmes for qualified nurses changed radically with the university degree pathway, even though the professional cultural evolution and reforms in Italian nursing studies had begun in 1992 (Palese, 2010). Currently, nursing training takes place at the university level (three-year cycle) and includes academic courses and practical internships; at the end of this, candidates are required to obtain a national licence. Some nurses continue with one-year or two-year specialisation programmes in fields such as public health, paediatrics, mental health, psychiatry and geriatrics, attending post-basic educational training. A Master of Science in Nursing has existed since 2004. Doctoral programmes have been available as well since 2006 (Rocco *et al.*, 2014).

The competencies of the Italian nursing staff have increased remarkably over the last 20 years.

Nevertheless, the contractual forms of advanced roles and competencies in nursing remain largely unrecognised, and the Italian organisational structure has remained unchanged: post-training or qualifications are not required to work as a nurse in specific fields in the INHS.

The system has remained doctors-centred, as is evident in all INHS reforms from its institution since the present (Ferré et al., 2014), even if PHC reforms have moved towards a more team-based approach (Fattore *et al.*, 2009; Armeni *et al.*, 2014; Seghieri *et al.*, 2014). On the other hand, it is known that collaboration with nurses, especially for chronic care, has the potential to improve the quality of care provided by GPs by allowing them to focus more on their own specific tasks while shifting the follow-up activities and the monitoring of therapy adherence to nurses.

## A mixed method investigation via a parallel concurrent design

A mixed method investigation via a parallel concurrent design (Creswell and Plano Clark, 2011) to identify the organisational models for the FCHN was conducted across Italy (Romero-Collado *et al*., 2017). A web survey was carried out on the National Federation of IPASVI Colleges (the Italian regulatory body for nursing) website, aimed at gathering opinions from nurses already working in the primary health system or who had attended post-basic training in public health. At the same time, some regional health plans and regional legislation concerning family nursing and national studies in primary health nursing were analysed. Eight Italian regions (Emilia Romagna, Friuli Venezia Giulia, Lazio, Lombardy, Piedmont, Puglia, Tuscany and Valle d’Aosta) were selected for analysis because they had a record of promoting family health nursing as part of their PHC systems. Relevant stakeholders (138) with different backgrounds (namely, regional political leaders, GPs, nurses, nurse managers, informal caregivers) were directly interviewed by the researchers using a semi-structured questionnaire to obtain a deeper understanding of the organisational models, activities and services provided, as well as to gauge patients’ and families’ level of satisfaction regarding family health nursing care.

## The opinions of the nurses responding to the web survey

Our web survey attracted 2204 respondents. We excluded those with >80% missing data, as well as 11 who declared, in an open question, that they were not nurses. The final sample on which the analyses were performed amounted to 1817 cases. The questionnaire consisted of 33 questions in 5 sections: (1) training; (2) professional and working context; (3) professional activity and experience; (4) role of the family nurse; and (5) socio-demographic data.

About two-thirds of the web survey respondents were nurses employed in district agencies, while one-third of the respondents were nurses who, at the time of the survey, had completed or were in the process of completing a post-basic training course in family and community health nursing or a similar programme. A considerable number of nurses had completed or were attending post-basic training in family and community health nursing while working in the hospital setting.

About 80% of respondents stated that they often worked with patients over 65 years of age, and mainly assisted people with diabetes, heart disease, COPD, cancer or ischemia.

The nurse respondents reported high levels of autonomy, especially in health status and care needs assessment and the planning and implementation of assistance to individuals.

Most of the nurses who attended post-basic FCHN courses were satisfied with their training programme, both in terms of training experience and skills acquisition. This demonstrates that the nurses who responded to the web survey recognised the benefits linked to expertise gained from additional training.

The competencies of primary interest were those which broadened the field of action, particularly those which represented a bridge between either family health physicians and families, or families and services or health institutions. Extremely relevant were competencies associated with ‘identifying and assessing the health status and health needs of individuals and families within the context and the culture’, ‘giving advice on lifestyle and behavioural risk factors’, ‘teaching families and individuals to recognise signs and symptoms’ and ‘helping individuals and families to cope with illness, chronic disability and stress’.

The FCHN was considered quite relevant by 43.3% and very relevant by 47.3% of the respondents with respect to reducing the number of inappropriate accidents and emergency hospital admissions. Likewise, the FCHN was regarded as quite relevant by 44% and very relevant by 44.2% of respondents with respect to reducing overall hospital admissions, and quite relevant by 46.1% and very relevant by 45.8% with respect to decreasing hospital readmissions. Moreover, the FCHN was considered decisive for cost reductions in the INHS (36.6%, quite relevant; 53.6%, very relevant).

The FCHN allows for improvements in the primary care system through connections made between general medicine physicians and individuals/families, and between families and local health care, particularly due to enhancements in hospital–community continuity regarding early and/or complex discharge (quite relevant by 31.4% and very relevant by 64.4% of respondents).

In short, the impact of family and community health nursing is relevant for the transition away from the hospital-centric model and thus for the improvement in the care continuity of the Italian healthcare system.

Finally, the FCHN has a strong impact on both individuals and families.

The respondents thought that the FCHN should care for older individuals with chronic diseases and multiple pathologies. This opinion reflects the daily work of the Italian nurses in the district agencies, as described in the Italian literature (Sasso *et al.*, 2005; Pellizzari, 2008; Scalorbi, 2012).

The responses demonstrated that health professionals were aware of the skills, activities and health conditions that should be the responsibility of the FCHN, in a manner consistent with the guidelines set forth by the WHO European Region (2000).

In particular, our findings suggested that the FCHN with advanced competencies and autonomy, capable of assisting patients who today mainly comprise older individuals with chronic diseases, acting as a bridge between GPs and families, and families and health services, contributes to continuity of care as emphasised by the WHO European Region (2000). This positive outcome reinforces the idea that proposing the formal institution of the FCHN is possible in Italy, as has already occurred in such countries as Slovenia and Scotland, where the FCHN has been officially recognised (Hennessy and Gladin, 2006; Murray, 2008; Martin et al., 2013).

## The regional pilot model of the FCHN role

The second part of our investigation examined the regional situation of the FCHN role. A background analysis was conducted, which involved the examination of regional planning documents, regional legislation concerning family and community health nursing, and national studies in nursing applied to public health and primary care between 2004 and 2014. Emilia Romagna, Friuli Venezia Giulia, Lazio, Lombardy, Piedmont, Puglia, Tuscany and Valle d’Aosta were selected for deeper exploration because they presented specific planning information or regulatory provisions, or more generally, promoted the FCHN in primary care.

From this analysis, we found that the FCHN has been gradually introduced into regional PHC systems through a range of initiatives, legislative actions, deployments in local projects and experimental introductions into local healthcare agencies. Nonetheless, the role of the FCHN has not been well defined, and a lack of its formal recognition by means of specific forms of employment contracts is evident.

The most recent legislation shows that all the analysed regions have been engaged in reorganising the healthcare system, particularly PHC. In general, the situation is heterogeneous and complex in terms of both functions and service descriptions, as mentioned in the introduction of this paper.

There are situations where the FHN and the community health nurse (CHN) are considered completely overlapping roles, and cases where there is a marked difference. Thus, to identify nurses who perform functions similar to those of FHNs, different terms are used like ‘care manager’ (Puglia, Tuscany and Piedmont), ‘case manager’ (Emilia Romagna, Lazio), ‘community nurse’, ‘micro-area nurse’ (Friuli Venezia Giulia) and ‘primary nurse’ (Piedmont). Taking a distance from the definition of the FHN is linked to the desire to not be assimilated into doctors’ solo practice and to avoid conflict between nurses and physicians or any resistance by doctors in regard to an increase in nurses’ professional autonomy (Hojat *et al.*, 2003; Brown *et al.*, 2011).

The analysis allowed for the identification of two family and community nursing development models.

The first model refers to family nursing applied to a socially and community-defined context. This is the prevalent model and is characterised by the following:

1. The identification of a defined geographical area (with between 1500 and 3000 inhabitants);
2. The homogeneous targeting of assisted persons with respect to socially and economically defined criteria;
3. Direct contact with the population;
4. The presence of a trusting relationship between nurses and assisted persons;
5. A broader view of care as not only for the frail or chronically ill but also for the healthy; and
6. A strong system of district governance that supports the nurses’ activities.

The second model, in contrast, refers to nursing in the chronic care model (Bodenheimer *et al.*, 2002), which is applied in Tuscany and Puglia. In these two cases, patients were included in surveillance programmes, and a proactive approach towards people with chronic diseases (Tuscany) or at risk of chronicity (Puglia) was the nurses’ responsibility.

Furthermore, the appropriate patient-to-staff ratio for the FCHN was unclear. In the Italian regions analysed in our study, the ratio was between 1000 and 2500 inhabitants per nurse. In contrast, GPs have a limit, subject to certain exceptions, of 1500 adult patients (over 13 years), while paediatricians are limited to a maximum of 800 children (below 14 years); however, exceptions can be granted in certain local situations, as happens in cases where no other paediatricians in a specific area are available or when a new child is added to an already included family (Italian Ministry of Health, 2017).

## The proposals for the creation of the family and community nurse role

From the analysis briefly described above, it is possible to deduce the role of the FCHN in Italy, as professionals provided with their own skills and autonomy oriented towards providing care for people – mainly chronically ill and older people – through preventative measures and interventions, in close collaboration with GPs. In this way, nurses who did specific post-basic studies could take on some activities done by GPs, without forgetting that some activities are, inevitably, at the physician’s discretion.

For the formal institution of the FCHN in Italy, it is important to consider the following conditions:

1. Providing formal recognition of the role of the FCHN, as well as a specific contractual form, which should be accompanied by simultaneous recognition of other nursing specialisations and advanced competencies for caring in the PHC system;
2. Foreseeing the inclusion of the FCHN in the health services network;
3. Considering interprofessional conflict and overcoming restrictions on the prescription of devices and aids by nurses;
4. Establishing a clear staff-to-patient ratio.

Facing these issues requires an organisational review (organisational level); making political decisions, in terms of both professional and social policy (political level); and making theoretical reflections (theoretical level).

Below, each of these themes is discussed, starting with the organisational framework for the development of the FCHN role.

### Organisational level

**Figure 1.**
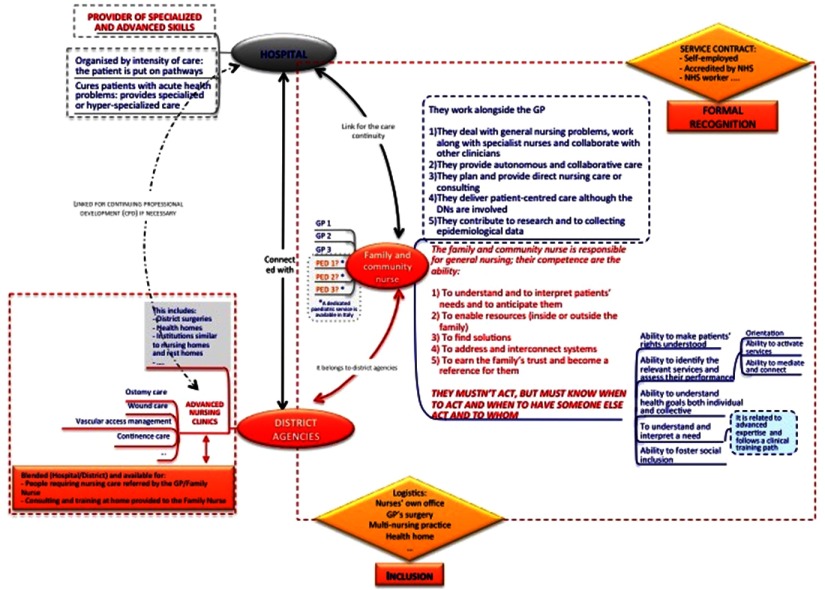
General framework for family and community nursing in Italy

The general organisational framework is represented in Figure 1.

Figure 1 shows the relation between institutions such as hospitals and district agencies and the possible role of the FCHN.

The FCHNs are embedded in district agencies, and in this way obtain inclusion in the health services network, avoiding the solo practice model, which has been recognised as causing governance problems in GPs’ activities. The FCHN can practise in-group, in nurses’ own offices, in GPs’ surgeries or in other locations, according to the specific regional or local organisation.

Formal recognition might occur through different contractual forms, such as self-employment accredited by the INHS or as a dedicated INHS worker.

It may be important to avoid having a 1:1 ratio between the FCHN and GPs or paediatricians because of the propensity of Italian physicians to consider nurses as their personal assistants or secretaries rather than as their partners (Vegesna *et al.*, 2016).

The patient is central as they choose their own FCHN in a relationship of trust. Therefore, the FCHN could set up a collaborative relationship with more than one GP or paediatrician.

The FCHN should work alongside GPs or paediatricians, and their principal activities should be:

1. Dealing with general nursing problems while working alongside specialist nurses and collaborating with other clinicians;
2. Providing autonomous and collaborative care;
3. Planning and providing direct nursing care or consultations;
4. Delivering patient-centred care even when other nurses who already work in district agencies are involved, such as delivering integrated home care (named Assistenza Domiciliare Integrata – ADI) (Ferré *et al.*, 2014); and
5. Contributing to research and epidemiological data collection.

The FCHN should be responsible for general nursing and have the following competencies:

1. Understanding and interpreting patients’ needs and anticipating them;
2. Enabling resources (inside or outside the family);
3. Finding solutions related to the health of assisted patients;
4. Addressing and interconnecting health and social systems;
5. Earning the family’s trust and becoming a point of reference for them.

The ability to recognise when the direct action of the FCHN is necessary, or when and who someone else has to act, is relevant. This means that the FCHN should not deliver all the care directly; in some cases, they should refer patients to specialist support (Martin *et al.*, 2013), while in others, non-specialist cases (eg, family members or social workers) should be referred to for informal support. In Italy, the phenomenon of so-called ‘Badanti’ (domestic caregivers) is increasing; these are informal paid carers, mostly immigrants (Ferré *et al.*, 2014), quite often, such carers do not have any formal training to provide appropriate care, and in such cases, the FCHN can play an important educational role, one that could prevent long-term problems.

In this framework, the hospital setting is configured as a provider of advanced and specialised clinical skills.

All the involved subjects, hospitals, district agencies and the FCHN are connected towards the continuity of care and continued professional development.

Some structures, such as district surgeries, healthcare homes and nursing homes, or community hospitals, belong to district agencies; while advanced nursing clinics specialising in, for example, ostomy care, wound care, vascular access maintenance and continence care, must be created with formal recognition of the nurses’ advanced competencies. These advanced nursing clinics should be available for people who require nursing care as referred by GPs/FCHN, as well as for the FCHN to consult with and train at home.

### Political level

Establishing a clear of FCHN-to-patient ratio is not just a professional aim, but a welfare policy as well. There is little knowledge and understanding about the workload and staffing levels (eg, staff-to-patient ratios) of the community nurse workforce (Kirby and Hurst, 2014), and benchmarks are influenced by elements like patient dependency, nursing activity and staff mix, which are in turn related to local policies and organisational decisions.

In Italy, problems regarding the governance of the health workforce are not only related to the number of nurses or other professionals; there is a more general and complicated quantitative (how many) and qualitative (what specialities, what roles) problem with planning as well (Vicarelli and Pavolini, 2015).

In Italy, 77.7% of the nurse workforce operates in hospital settings (CENSIS, 2017), which comes at the expense of the primary care workforce. At present, there is no nursing workforce model within primary care, as reported by the Royal College of Nursing (RCN) (2015), and studies showing that a better patient-to-nurse ratio could improve patient safety and organisation, as well as the quality of care (Sasso *et al.*, 2016), have been conducted mainly in hospital settings. Moreover, in Italy, a structured system for evaluating the quality of PHC is still lacking (Manzoli *et al.*, 2014). Therefore, estimating the right number of patients for each nurse is difficult.

**Figure 2.**
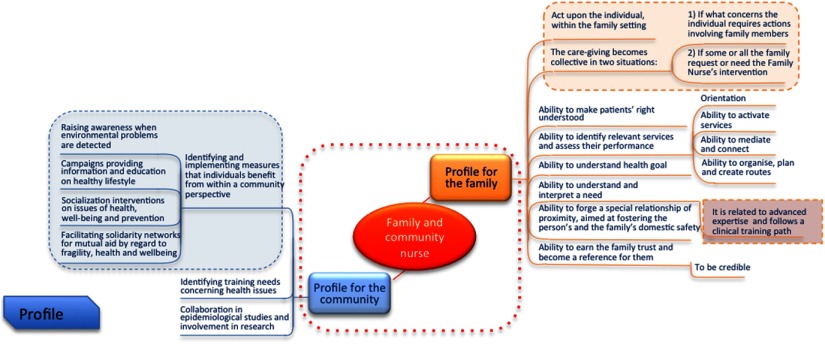
The two FHN and/or CHN profiles for families (micro-level) and communities (macro-level)

In our model, the FCHNs are an instrument for social innovation: they can foster better health as well as improved social outcomes, both at the micro-level (ie, individuals and families) and the macro-level (ie, communities). It is necessary for policymakers to evaluate and choose one or two different profiles (Figure 2) according to the local situation or resources available: FHN and/or CHN. Alternatively, the FCHN could be used to fulfil different roles depending on specific circumstances.

Each of these profiles may include a specific number of assisted persons or act as a reference for a specific area.

The problem of interprofessional conflict is related to two elements: collaboration and prescribing. Research exploring collaboration between GPs and nurses suggests that there are some problems yet to be solved (Hansson *et al.*, 2010; Sollami *et al.*, 2015).

In particular, it was shown that physicians tend to express a lesser inclination towards full collaboration and power sharing with nurses since historically they have been in the position of power, while nurses have a more positive view of collaboration overall. In the primary care setting, GPs may still view nurses as subordinates (Vegesna *et al.*, 2016). In our interviews, this viewpoint emerged in situations in which GP groups hired nurses with a direct contractual agreement. The employer-dependent relationship enhances the power of physicians in a hierarchical manner, limiting nurses’ autonomy.

Prescribing is the second area of debate in Italy. At present, the nursing field is evolving and services should be changed according to demand: specialist competencies require freedom from limits and boundaries that are based on an old vision of the relationship between the work of physicians and nurses.

In other countries, the right to prescribe medicines has been extended in recent years to other healthcare professionals, such as nurses or pharmacists (Cooper *et al.*, 2011). Nurses can legally prescribe medications in Australia, Botswana, Canada, Finland, Ireland, New Zealand, Norway, South Africa, Spain, Sweden, The Netherlands, UK, USA and Zimbabwe (Gielen *et al.*, 2014; Weeks *et al.*, 2016; Nuttall, 2017; Romero-Collado *et al.*, 2017).

In Italy, only physicians can prescribe medicines, aids and devices eligible for Italian National Health Care cover: supplementary non-medical prescribing (NMP) is not allowed. Prescription-free medicines, like over-the-counter medications (OCM), are not normally on display and people have to ask to the pharmacists because all the medicines are stored at the back of the pharmacies where only the pharmacists have access to them.

Italy has been recognised as a country with limited task shifting from physicians to nurses. Task-shifting has been identified as a strategy to improve quality and efficiency. Where task shifting is limited, advanced nursing practice encounters boundaries and is usually under physicians’ supervision (Maier and Aiken, 2016).

**Table:** The situation of Italian nursing prescribing is shown in the following SWOT analysis:

***S**trengths*	- Availability of nursing advanced educational programmes (Rocco *et al*., 2014).	- High numbers of physicians-to-population ratio (OECD, 2017).	***W**eaknesses*
	- Nurses’ technical knowledge in some fields is superior to other professionals – for example: wound care, ostomy care or continence care…(Marcadelli and Bertolazzi, 2017).	- Low nurse-to-doctor ratio (OECD, 2017).	
		- Over 77% of the nursing workforce operates in hospital settings (CENSIS, 2017).	
		- Absence of recognition of nursing specialisations (Rocco *et al.*, 2017).	
***O**pportunities*	- Collaboration with nurses, especially for chronic care, has the potential to improve the quality of care (Armeni *et al.*, 2014; Seghieri *et al.*, 2014).	- Italian physicians show resistance to autonomous nurse practices and they still view nurses as subordinates (Brown *et al.*, 2011; Vegesna *et al.*, 2016).	***T**hreats*
	- The international context is in favour of nurses prescribing within a part of task shifting as an improvement in quality and efficiency (Maier and Aiken, 2016).	- Nurse prescribing engenders conflict and disagreement with physicians (Cooper *et al.*, 2011)	
	- Nurse prescribing has a positive impact on several elements such as integrating care within a holistic approach, better quality of care, service benefits (Nuttall, 2017).		

At present, the request for prescribing nurses in Italy could harm the development of the nursing profession and simply create further interprofessional conflict. Nonetheless, the fact that the international context reflects the need for prescribing nurses may be an important enabler to the development of Italian nurses’ competencies. More realistically, the implementation of the prescribing nurse could start with the prescription of aids and devices, which are normally part of the nurses’ daily activities.

In the opinion of the interviewees, the prescription of aids and devices is an area where nurses are recognised as having specific expertise. Some of the interviewed physicians stated that nurses’ technical knowledge in wound care, for example, is superior to that of other professionals and needs to be recognised (Marcadelli and Bertolazzi, 2017).

### Theoretical level

Two elements are relevant to this theme: the role of nurses as systemic connectors, and a two-way relationship with patients and families.

**Figure 3.**
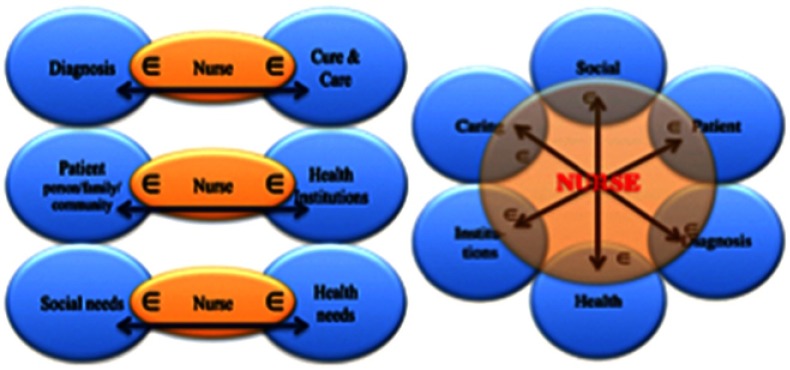
The sharing of paradigms with other social and health professional sciences and nurses as systemic connectors

Systemic connection is a potentially powerful role for nurses (Marcadelli, 2016). Nursing science shares paradigms with other social and health professional sciences (see Figure 3, in which the sharing of paradigms is indicated with the mathematical symbol **∈**, which represents ‘belongs to’).

Nurses are professionals who can both understand the language of diagnosis and care and translate it for the assisted persons and their families to obtain mutual comprehension and shared meaning. Moreover, nurses can link assisted persons, families and communities to health institutions because they know where and how to ask for services/help.

**Figure 4.**
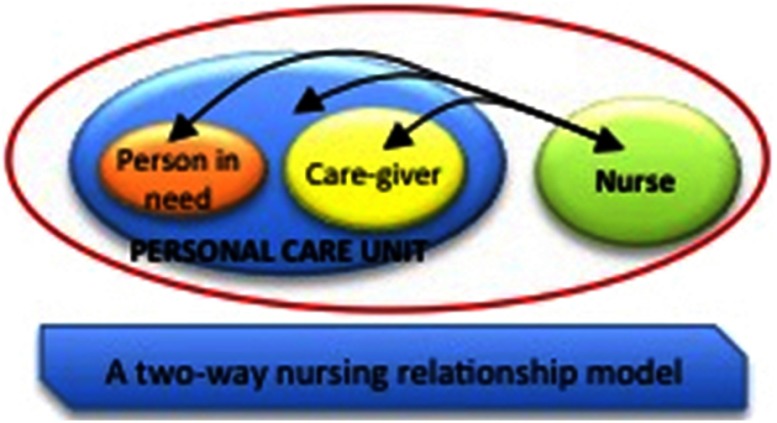
Theoretical model for nursing care: A two-way relationship model

Last, but certainly not least, nurses can assess and recognise social or health needs, and can refer assisted persons, families and communities to appropriate agencies or request appropriate professional intervention. A vision of nurses’ actions according to the model is shown in Figure 4, in which nursing care, which is a relationship also comprising techniques, winds up in a variable action field passing from the individual to the caregiver to both as needed. In the model, the position of the nurse ‘adjacent to’ and not ‘above’, the patient and family highlights the willingness to be in a less asymmetric relationship, but in a meaningful partnership (Marcadelli and Artioli, 2010).

Obviously, the nurse does not act alone, but carries out interventions autonomously or collaboratively while being included in community teams.

Professional and political strategies should be oriented towards investing in the FCHN because more than any other professional they can promote an authentic image of professional nursing. In addition, they can offer the following:

1. reaching out to the population and involving them, promoting participation and information initiatives;
2. being recognised as proximity reference;
3. acting as systemic connectors towards understanding different worlds (diagnosis and treatment, social needs and healthcare needs, individuals and institutions) to interpret of patients’ demands, which are often presented as non-specific, this is a field peculiar to nursing care, both in terms of training and objectives;
4. offering their competencies to the social and political worlds as concrete options for collective well-being and social innovation; and
5. providing their expertise in terms of reading and interpreting needs, without believing that interlocutors should be exclusively health professionals.

## Conclusions: Family and community nurses as an element for social innovation

**Figure 5.**
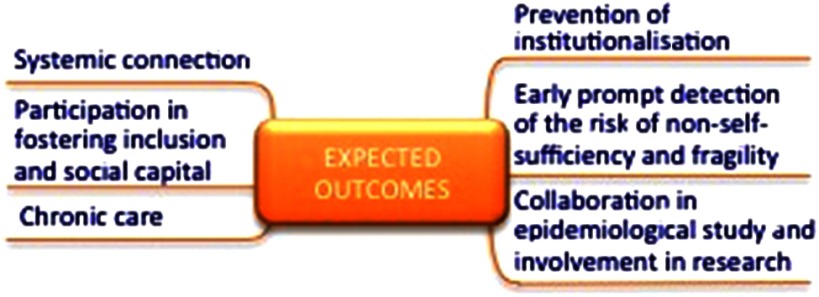
The expected outcomes from FCHNs

From this framework, which considers the organisational, the political and the theoretical levels, the expected outcomes for the FCHN role can be summarised within activities that have social value, carried out on individuals or groups (Figure 5). These activities include the prevention of institutionalisation, involvement in the creation of social inclusion and social capital development, early identification of the risk of non-self-sufficiency and the prevention of the acceleration of chronic diseases or their prompt detection (Liotta *et al.*, 2016). Together, these activities realise social innovation.

Social innovation is vital for new ideas that are able to simultaneously satisfy social needs while creating new social relationships (Murray *et al.*, 2010). At present, social innovation is all the more crucial, as we are witnessing growing social and health demands alongside strong constraints on public spending, and innovation is required for the social health system and welfare in general. Social innovation is not necessarily an answer provided in terms of new services but can include both a new mode of service delivery and solutions for people’s needs, as well as different roles for public or health service operators. New ideas can promote measurable and recognisable values in areas such as quality of life, solidarity and social welfare, as well as in the creation of collaborative relationships (Siza, 2015). Based on our proposal and analysis of regional experiences, we believe that family and community health nursing has the potential to provide such social innovation.
